# The complete chloroplast genome sequence of *Knema furfuracea* (Myristicaceae)

**DOI:** 10.1080/23802359.2019.1703570

**Published:** 2019-12-18

**Authors:** Tian Yang, Chang-Li Mao, Xiao-Qin Li, Feng-Liang Zhang, Qi Zhao, Yu Wu

**Affiliations:** Yunnan Institute of Tropical Crops, Jinghong, China

**Keywords:** *Knema furfuracea*, chloroplast genome, myristicaceae

## Abstract

*Knema furfuracea* is a member of Myristicaceae. The *K. furfuracea* chloroplast genome is found to be 154,527 bp in length and has a base composition of A (29.99%), G (19.31%), C (19.92%), and T (30.78%). The genome contained two short inverted repeat (IRa and IRb) regions (48,110 bp) which were separated by a large single copy (LSC) region (86,188 bp) and a small single copy (SSC) region (20,229 bp). The chloroplast genome has 87 protein-coding genes, 27 transfer RNA (tRNA) genes, and 8 ribosomal RNA (rRNA) genes. Further, complete chloroplast sequence of *K. furfuracea* was aligned together with two species of Myristicaceae and five basal angiosperms species which have reported the complete chloroplast sequence. This complete chloroplast genome will provide valuable information for the development of DNA markers for future species resource development and phylogenetic analysis of *K. furfuracea*.

*Knema furfuracea*, belongs to Knema of Myristicaceae, is a tall arbor tree and mainly distributed in Yunnan, China (Editorial Committee of Chinese Academy of Sciences Flora 1979). The bark and seeds of *K. furfuracea* are mainly used in medicine (Perry and Metzger 1980), So far, it has been analyzed on chemical constituents (Kuang 2019) fatty acid composition (Wu et al. 2015) and leaf epidermal morphology (Zhang and Xu 2010). In this study, we characterized the complete chloroplast genome sequence of *K. furfuracea* for phylogenetic analysis. The annotated genome sequence has been deposited Genbank under the accession number MK285563.

The fresh leaves of *K. furfuracea* was collected in 2017 from Lancang River valley, Yunnan, China (100°02.39′E, 21°13.77′N), at the same time, we also took the seeds and brought them back to the base, its seedlings are planted and preserved in specimen plantation of Yunnan Institute of Tropical Crops(YITC) and the specimen number is 20140615. The genome DNA of *K. furfuracea* was extracted using the DNeasy Plant Mini Kit (QIAGEN, Valencia, CA), and its remaining DNA was stored in an ultra-low temperature freezer now. Genome sequencing was performed using Roche/454, sequencing libraries were prepared by the GS Titanium library preparation kit. The chloroplast genome assembled using CLC Genomic Workbench v3.6 (http://www.clcbio.com). The genes in the chloroplast genome were predicted using the DOGMA program (Wyman et al. 2004).

The circular genome is 154,527 bp in size, and comprises a large single copy (LSC) region (86,188 bp), a small single copy (SSC) region (20,229 bp), and two short inverted repeat (IRa and IRb) regions (48,110 bp). The base composition of the circular chloroplast genome is A (29.99%), G (19.31%), C (19.92%), and T (30.78%). GC content of 39.23% for the whole *K. furfuracea* chloroplast genome. The *K. furfuracea* chloroplast genome has 87 protein-coding genes, 27 transfer RNA (tRNA) genes, and 8 ribosomal RNA (rRNA) genes. There were 30 genes duplicated in the IR regions. The LSC region contained 80 genes, which including 62 protein-coding genes and 16 tRNA genes whereas 6 protein-coding genes 3 tRNA genes and 4 rRNA genes were including in the SSC region. The introns were detected in 10 genes include *rpoB, psbB, atpH, rpl23, rps19-fragment, trnQ-UUG, trnS-GGA, trnV-GAC, ndhH, trnL-CAA* and they all have 1 intron.

To study *K. furfuracea* phylogenetic relationship with the angiosperms, *Horsfieldia pandurifolia* and *Myristica yunnanensis* of Myristicaceae (Changli et al. 2019a, 2019b) and other complete chloroplast genome sequences of angiosperms were download for analyses. The maximum likelihood phylogenetic was performed using MEGA X (Kumar et al. 2018) (Figure 1). A bootstrap analysis was performed on the resulting phylogenetic tree, and values were obtained after 1000 replications. The result shows that *K. furfuracea* was clustered with other species and closely to *Horsfieldia pandurifolia* and *Myristica yunnanensis* chloroplast complete genome.

**Figure 1.**
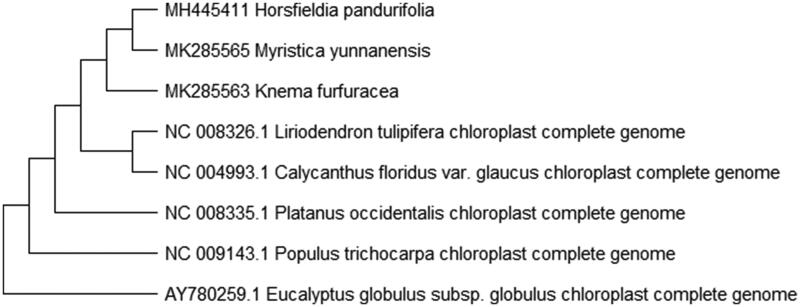
Maximum likelihood phylogenetic tree of *K. furfuracea* with seven species based on complete chloroplast genome sequences. The gene’s accession number is list in figure and the data of *H. pandurifolia* and *M. yunnanensis* come from author.

The complete chloroplast genome of *K. furfuracea* would provide information on development of molecular markers and phylogenetic analysis in the future.
